# Prevalence and Clinical Patterns of Central Post-stroke Pain Syndrome After Acute Ischemic Stroke: A Prospective Observational Cohort Study

**DOI:** 10.7759/cureus.114769

**Published:** 2026-08-18

**Authors:** Paras Gulati, Sravan Kumar Sara, Sandhya Manorenj, Mohammed Zoheb

**Affiliations:** 1 Neurology, Deccan College of Medical Sciences, Hyderabad, IND; 2 Neurology, Kerala Institute of Medical Sciences (KIMS) HEALTH, Trivandrum, IND

**Keywords:** cohort study, ischaemic stroke, neuropathic pain, post-stroke pain, sensory impairment, stroke complications

## Abstract

Background: Central post-stroke pain syndrome (CPSP) is a major neuropathic consequence after stroke, but the prevalence and clinical pattern are still inconsistently recorded. This study aims to investigate the prevalence, onset, and clinical presentation of CPSP after mild acute ischemic stroke.

Methods: We performed a prospective, hospital-based, observational cohort study of acute ischemic stroke patients. Development of CPSP was evaluated by clinical history, neurological examination, sensory profile, and neuroimaging correlation. Demographic variables, stroke characteristics, vascular risk factors, and functional outcome measures were analyzed.

Results: Of 720 acute ischemic stroke patients prospectively screened, 100 (13.9%, 95% CI 11.4-16.4%) met diagnostic criteria for CPSP and were included in the analysis. The mean age was 64.8 ± 7.1 years, and 63 (63.0%) were male. Middle cerebral artery territory lesions were most common (43.0%), followed by thalamic lesions (21.0%). Pain was contralateral to the stroke lesion in 98.0% of patients. In multivariable logistic regression, a higher NIHSS (National Institutes of Health Stroke Scale) score independently predicted poorer functional outcome (modified Rankin Scale (mRS) 2) at follow-up (OR: 2.915, 95% CI: 1.525-5.573, p = 0.001).

Conclusion: CPSP is a clinically significant complication after acute ischemic stroke and should be actively screened during follow-up. Early recognition can improve symptom management and rehabilitation outcomes.

## Introduction

Central post-stroke pain syndrome (CPSP) is a neuropathic pain syndrome that develops as a result of damage to the central nervous system following a cerebrovascular event. It is characterized by altered somatosensory perception with burning pain, dysesthesia, allodynia, hyperalgesia, and abnormal responses to non-painful stimuli [1,2]. Unlike the broader category of post-stroke pain, CPSP refers specifically to pain from central somatosensory pathway dysfunction, rather than musculoskeletal or peripheral causes [3].

Post-stroke pain is a clinical problem caused by many factors and could include shoulder pain, spasticity-related pain, myofascial pain, complex regional pain syndrome, and central neuropathic pain [3,4]. Of these, CPSP is one of the most difficult to recognize and treat, as the symptoms are often delayed, variable in presentation, and difficult to differentiate from other pain syndromes [5,6]. As a result, this condition is frequently missed during routine stroke follow-up.

The reported prevalence of CPSP in the literature varies widely, reflecting differences in study design, patient selection, type of stroke, timing of assessment, and diagnostic criteria [7-9]. CPSP is classically associated with thalamic lesions, but it is also seen after injury to the brainstem, thalamocapsular region, cortical sensory areas, or other areas involved in sensory processing [10-12]. The broader anatomical distribution suggests the syndrome is related to disruption of central pain pathways as opposed to isolated thalamic injury [13,14]. CPSP is relevant to health-related quality of life, sleep, daily activities, mood, and post-stroke rehabilitation [15]. Early identification is essential because persistent pain can interfere with recovery and decrease adherence to rehabilitation [2].

We therefore designed this prospective observational cohort study with a twofold objective: to establish the prevalence of CPSP among acute ischemic stroke patients screened during the study period and to characterize the temporal profile, sensory features, lesion location, and clinical/functional correlates of CPSP among patients meeting diagnostic criteria. Appreciation of these features may improve recognition and guide more effective management strategies in stroke survivors.

## Materials and methods

Study design and context

This prospective observational cohort study was carried out in the Department of Neurology, Deccan College of Medical Sciences, Hyderabad, Telangana, India, for a period of two years from June 2024 to June 2026. The study aimed to assess the clinical profile and patterns of central CPSP in patients with acute ischemic stroke during follow-up after discharge from the hospital.

Study group

We screened adult patients (≥18 years old) who had a confirmed acute ischemic stroke on brain magnetic resonance imaging (MRI) during the follow-up visits in the outpatient department beginning two weeks after discharge. Eligible patients had mild stroke-related disability and severity, defined by a modified Rankin Scale (mRS) score of 0-2 [16] and a National Institutes of Health Stroke Scale (NIHSS) score of less than 5 [17], and were required to give informed consent for participation.

Participant flow

**Figure 1:**
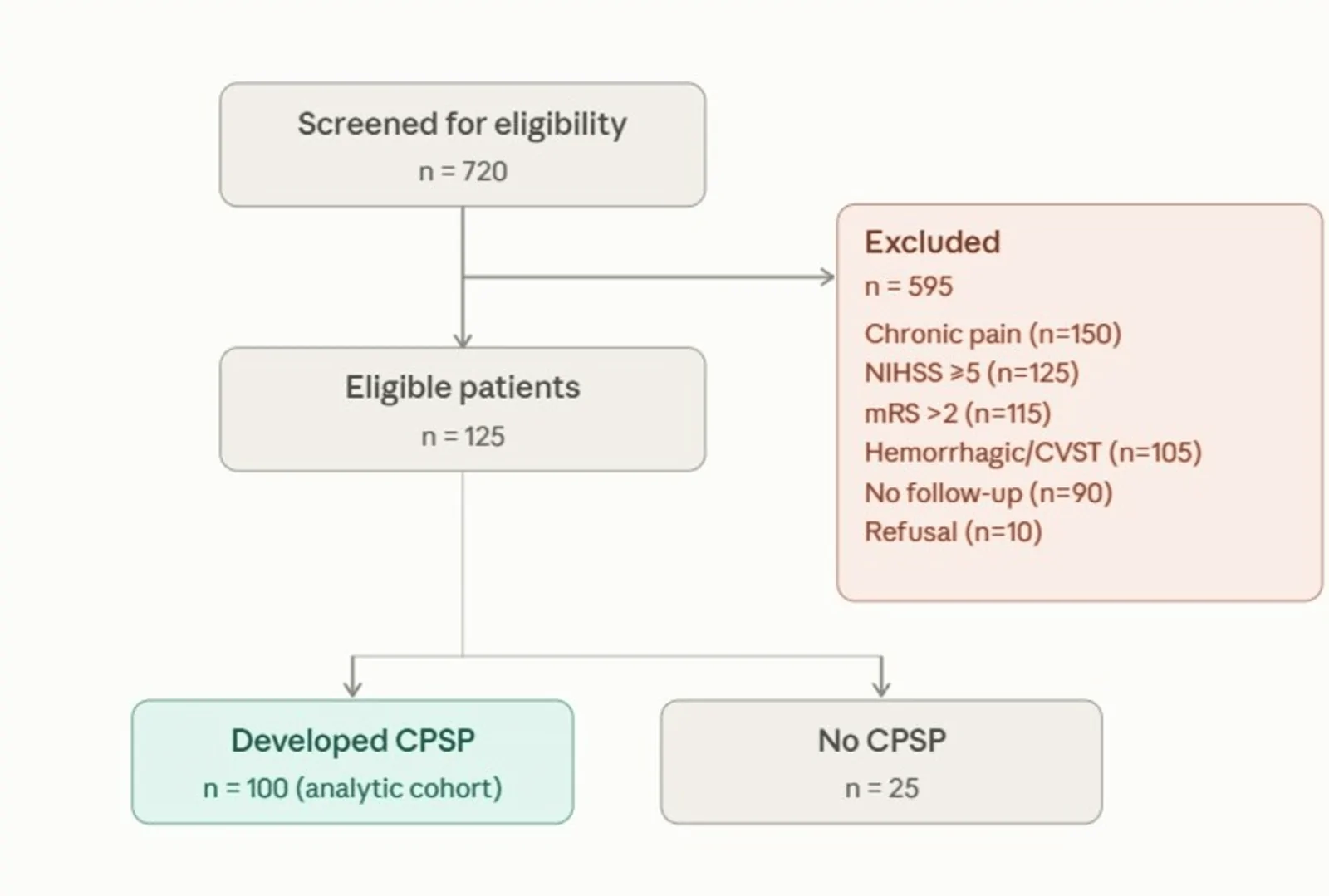
Patient flow diagram NIHSS: National Institutes of Health Stroke Scale; mRS: modified Rankin Scale; CVST: cerebral venous sinus thrombosis; CPSP: central post-stroke pain syndrome

Of 720 acute ischemic stroke patients screened during the two-year study period, 105 were excluded for hemorrhagic stroke or cerebral venous sinus thrombosis, 125 for NIHSS ≥5, 115 for mRS >2, 150 for pre-existing chronic pain prior to stroke onset, 10 for refusal to participate, and 90 for absence of follow-up beyond two weeks. Of the remaining 125 eligible patients, 100 met diagnostic criteria for CPSP and constitute the analytic cohort reported here; the remaining 25 eligible patients did not develop CPSP during the observation period. A participant flow diagram summarizing this selection process is provided as Figure 1.

Inclusion and exclusion criteria

Inclusion criteria were adult patients (≥18 years of age) with MRI-confirmed acute ischemic stroke, follow-up after at least two weeks of discharge, mRS score of 0-2, NIHSS score of less than 5, and willingness to provide informed consent. This severity range was selected because patients with mild stroke-related disability are generally able to reliably self-report sensory and pain symptoms without confounding from major cognitive, language, or motor impairment, allowing more accurate clinical characterization of CPSP features in this subgroup. Exclusion criteria were age under 18 years, hemorrhagic stroke, cerebral venous sinus thrombosis, pre-existing chronic pain before stroke onset, NIHSS score above 5, mRS score above 2, refusal to participate, or absence of follow-up after two weeks.

Clinical assessment

Patients were evaluated using a standardized worksheet, questionnaire, and follow-up assessment pro forma designed to identify pain, discomfort, or unpleasant sensations persisting after stroke. Pain assessment included the anatomical location and laterality of pain, the time of onset after stroke, and associated sensory symptoms, as well as analgesic use. Functional dependence and disability were assessed during follow-up using the mRS, while stroke severity was assessed using the NIHSS. All clinical and pain assessments were performed by two trained study neurologists using the standardized worksheet, with joint case review and consensus for the diagnostic classification of CPSP. Formal interobserver agreement was not separately quantified in this study, which is acknowledged as a limitation; disagreements in diagnostic classification were resolved by consensus discussion among the assessing neurologists. Each patient underwent a single structured outpatient assessment, conducted no earlier than two weeks after hospital discharge; the actual interval from stroke onset to assessment varied across patients (median 51 days, IQR 33-65) and was recorded as a study variable, but patients were not followed serially with repeat assessments over time.

MRI protocol

Brain MRI was performed at the time of acute admission using a 1.5-Tesla scanner, with diffusion-weighted imaging (DWI) and all standard stroke sequences acquired. Each scan was reported by a radiologist and independently reviewed and confirmed by two experienced neurologists to determine the stroke territory. Lesion territory (middle cerebral artery (MCA), thalamic, posterior cerebral artery (PCA)/occipital, capsular/corona radiata, brainstem/pons, medullary, or frontal) was assigned based on this consensus radiological-neurological review.

Definition and diagnostic approach

CPSP was diagnosed according to the IASP/NeuPSIG (International Association for the Study of Pain/Neuropathic Pain Special Interest Group) criteria for central neuropathic pain: (1) pain with a distribution that is neuroanatomically plausible given the stroke lesion; (2) a history suggestive of a central nervous system lesion; (3) demonstration of a relevant central lesion by neuroimaging, or a positive sensory examination confined to the painful area; and (4) exclusion of other causes of pain (musculoskeletal, spasticity-related, complex regional pain syndrome, or pre-existing pain) by history and examination. Laterality of pain, associated sensory abnormalities (allodynia, hyperalgesia, thermal dysesthesia), and lesion location on MRI were recorded to establish clinicoradiological correlation.

Study parameters

Demographic and clinical variables included age, sex, vascular risk factors, stroke territory, TOAST subtype [18], lesion laterality, pain laterality, NIHSS score, mRS score, and interval from stroke onset to follow-up assessment. Stroke territory was categorized from neuroimaging findings into MCA, thalamic, PCA/occipital, capsular/corona radiata, brainstem/pons, medullary, and frontal involvement in the analyzed cohort.

Sample size estimation

The minimum required sample size was estimated using Cochran's formula for estimating a population proportion:

$n_0=\frac{Z^2\ \ \times\ \ p\left(1-p\right)}{d^2}$

where Z = 1.96, corresponding to a 95% confidence interval; p = 0.5, representing the assumed proportion in the absence of prior prevalence data (this value maximizes the required sample size and is therefore the conservative standard choice); and d = 0.05, the acceptable margin of error.

$n_0=\frac{\left(1.96\right)^2\times0.5\left(1-0.5\right)}{\left(0.05\right)^2}=384.16\approx385\text{}$

As the source population was finite, comprising N = 720 stroke patients screened over the two-year study period, the finite population correction (FPC) was applied to adjust the initial estimate:

$n_{adj} = \frac{n_0}{1 + \dfrac{n_0 - 1}{N}} = \frac{385}{1 + \dfrac{384}{720}} \approx 251$

Although the statistically calculated sample size requirement was approximately 251, CPSP is a relatively uncommon sequela of stroke, with reported incidence rates of approximately 8-10.5% among stroke patients [19]. Consequently, of the 720 stroke patients screened, only 100 patients met diagnostic criteria for CPSP and were available for inclusion in the final analysis. Because the entire finite source population (N = 720) was screened, this shortfall reflects the true, expected rarity of CPSP in this cohort rather than incomplete recruitment, but it nonetheless limits statistical power for the subgroup and regression analyses reported below. This shortfall relative to the calculated requirement represents a limitation of the study and is acknowledged accordingly; findings should therefore be interpreted as preliminary, with adequately powered future studies warranted to confirm these observations.

Statistical analyses

The data were entered into a secured database and analyzed using IBM SPSS Statistics for Windows, Version 23 (Released 2015; IBM Corp., Armonk, New York). Continuous variables are expressed as mean ± standard deviation or median and interquartile range; categorical variables are expressed as frequency and percentage. Group comparisons were performed using t-tests and chi-square tests described in the study protocol. The analyzed results included Welch t-tests, Mann-Whitney U tests, chi-square or Fisher exact tests, and multivariable logistic regression with mRS 2 as the dependent outcome variable. Candidate variables for the multivariable model (NIHSS, age, sex, hypertension, diabetes mellitus, thalamic stroke, and right-sided stroke) were pre-specified a priori based on clinical relevance and prior literature, rather than selected through univariable screening or stepwise methods. The final analytic cohort (n=100) had complete data for all variables included in the regression model, and no imputation was required. A p-value < 0.05 was taken as statistically significant.

Ethics statement

The study was carried out according to the ethical principles of the Declaration of Helsinki. Institutional ethics committee approval was obtained prior to the initiation of the study, and written informed consent was obtained from all participants or their carers before recruitment. The final analytical dataset for the present manuscript consisted of patients with post-stroke central pain symptoms registered in the master chart, and internal comparisons were made within this group.

## Results

Of 720 acute ischemic stroke patients screened, 100 (13.9%, 95% CI 11.4-16.4%) met diagnostic criteria for CPSP and were included in the analysis. The mean age was 64.8 ± 7.1 years; 63 (63.0%) were male; hypertension was present in 89 (89.0%); and diabetes mellitus in 67 (67.0%). The mean mRS was 1.39 ± 0.60, the mean NIHSS was 3.52 ± 0.77, and the median interval from stroke onset to follow-up was 51 days (IQR: 33-65) (Table 1).

**Table 1:** Baseline characteristics of the study population NIHSS: National Institutes of Health Stroke Scale; mRS: modified Rankin Scale; IQR: interquartile range

Characteristic	Value
Number of cases	100
Age, mean ± SD (years)	64.8 ± 7.1
Age, median (IQR)	65 (59–69)
Male sex, n (%)	63 (63.0%)
Hypertension, n (%)	89 (89.0%)
Diabetes mellitus, n (%)	67 (67.0%)
mRS, mean ± SD	1.39 ± 0.60
NIHSS, mean ± SD	3.52 ± 0.77
Follow-up lag, median (IQR) days	51 (33–65)

MCA territory involvement was the most common lesion pattern, accounting for 43.0% of cases, followed by thalamic lesions in 21.0%, PCA/occipital lesions in 14.0%, and capsular/corona radiata lesions in 13.0%. Brainstem/pons and medullary lesions each accounted for 4.0% of cases, while frontal lesions accounted for 1.0% (Table 2).

**Table 2:** Stroke territory distribution in the study cohort MCA: middle cerebral artery; PCA: posterior cerebral artery

Territory	n	%
MCA	43	43.0
Thalamic	21	21.0
PCA/occipital	14	14.0
Capsular/corona radiata	13	13.0
Brainstem/pons	4	4.0
Medulla	4	4.0
Frontal	1	1.0

**Figure 2:**
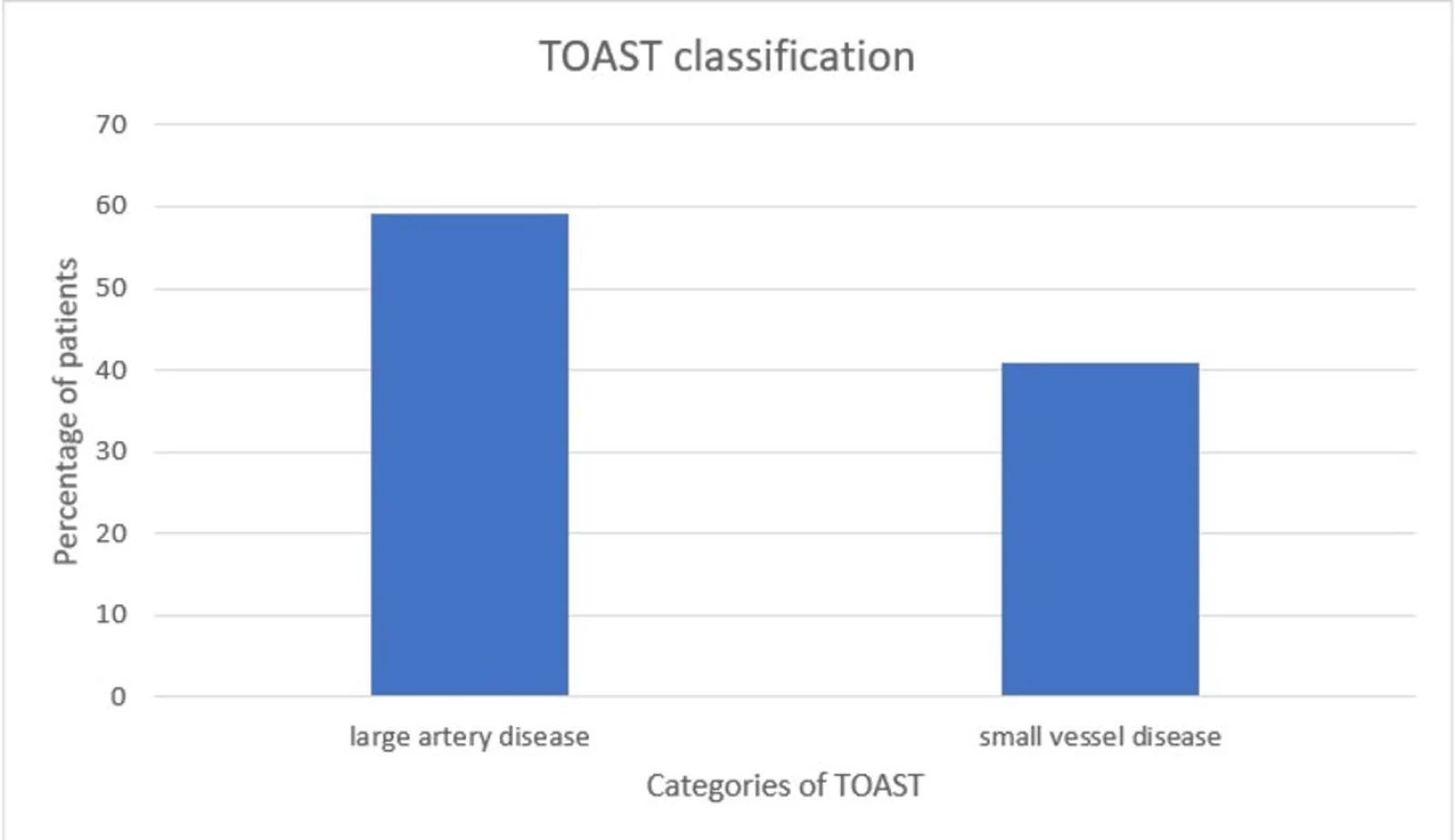
TOAST subtype distribution in the study population

Based on the TOAST classification, 59.0% of patients had large-artery disease, and 41.0% had small-vessel disease (Figure 2).

**Figure 3:**
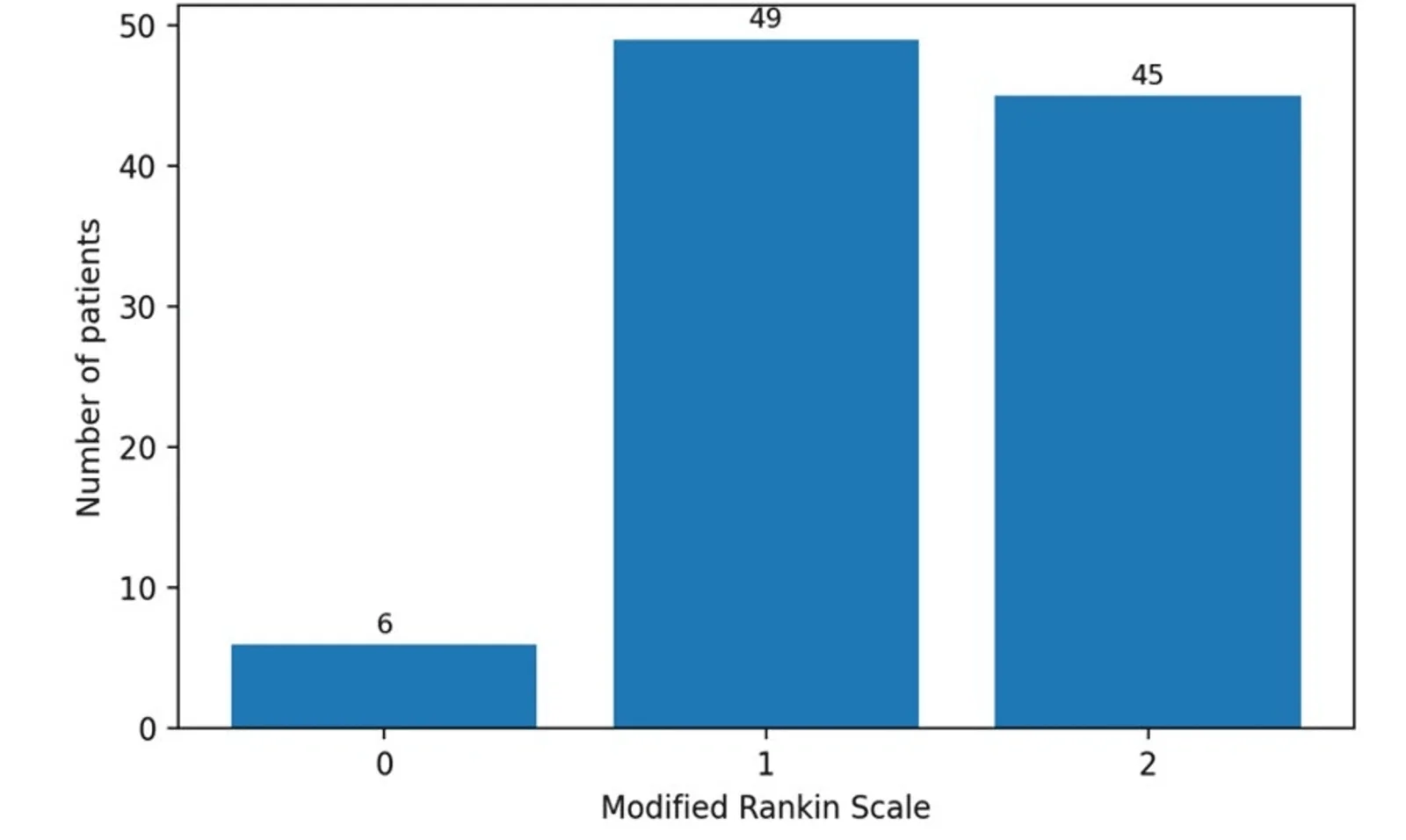
Distribution of modified Rankin Scale scores in the study population

**Figure 4:**
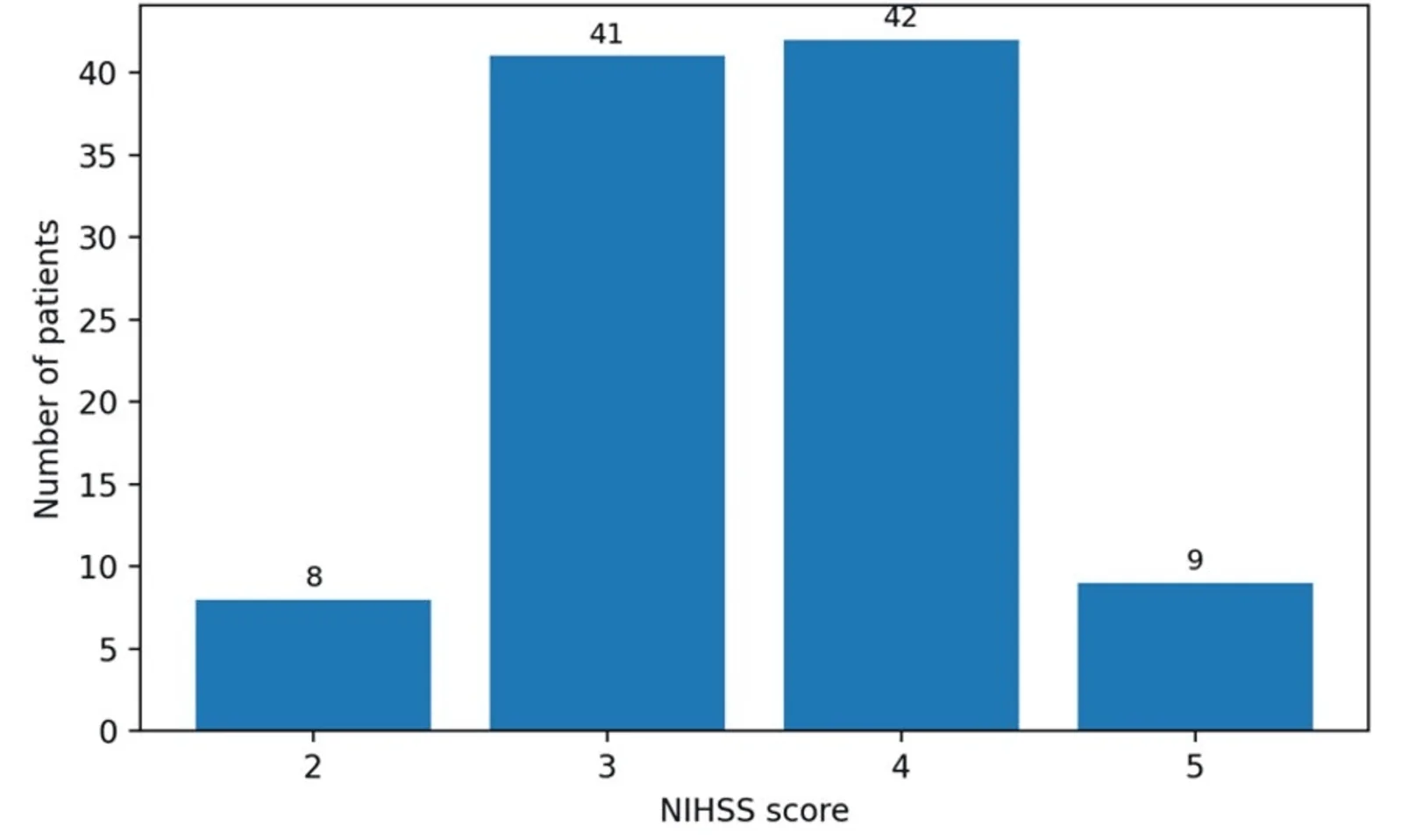
Distribution of NIHSS scores in the study population NIHSS: National Institutes of Health Stroke Scale

At follow-up, mRS scores were 0 in 6.0% of patients, 1 in 49.0%, and 2 in 45.0%. NIHSS scores were 2 in 8.0%, 3 in 41.0%, 4 in 42.0%, and 5 in 9.0%, indicating that most patients had mild stroke severity. Pain was contralateral to the stroke lesion in 98.0% of patients (Figures 3, 4).

Patients with thalamic lesions had significantly higher NIHSS scores than those with non-thalamic lesions (3.81 ± 0.68 vs. 3.44 ± 0.78, p = 0.041), while no significant differences were observed for age, mRS, follow-up interval, sex, hypertension, or diabetes mellitus. Patients with mRS 2 had significantly higher NIHSS scores than those with mRS 0-1 (3.80 ± 0.69 vs. 3.29 ± 0.76, p = 0.001), with no significant differences in age, sex, hypertension, diabetes mellitus, or thalamic stroke frequency (Table 3).

**Table 3:** Thalamic vs. non-thalamic stroke: demographic and clinical comparison * Statistically significant NIHSS: National Institutes of Health Stroke Scale; mRS: modified Rankin Scale; IQR: interquartile range

Variable	Thalamic (n=21)	Non-thalamic (n=79)	p-value
Age, mean ± SD	64.48 ± 6.22	64.92 ± 7.36	0.780
Lag days, median (IQR)	61 (37–65)	48 (33–64)	0.184
mRS, mean ± SD	1.19 ± 0.68	1.44 ± 0.57	0.129
NIHSS, mean ± SD	3.81 ± 0.68	3.44 ± 0.78	0.041*
Male sex, n (%)	15 (71.4)	48 (60.8)	0.451
Hypertension, n (%)	20 (95.2)	69 (87.3)	0.450
Diabetes mellitus, n (%)	15 (71.4)	52 (65.8)	0.795

**Figure 5:**
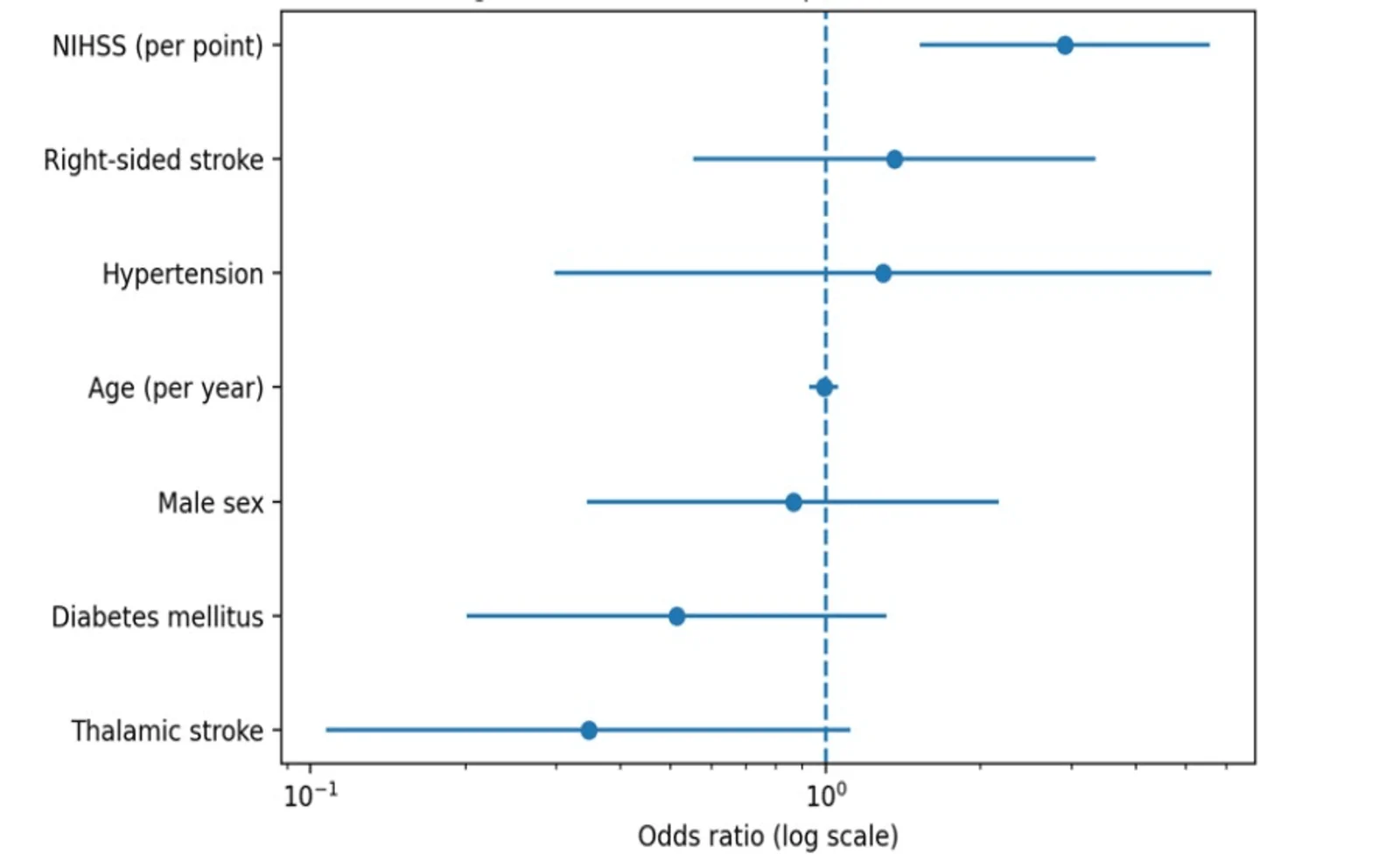
Multivariable logistic regression for mRS 2 NIHSS: National Institutes of Health Stroke Scale; mRS: modified Rankin Scale

In multivariable logistic regression, higher NIHSS independently predicted mRS 2 at follow-up (OR: 2.915, 95% CI: 1.525-5.573, p=0.001). Thalamic stroke showed a borderline inverse association with mRS 2 (OR: 0.346, 95% CI: 0.107-1.117, p=0.076), whereas age, sex, hypertension, diabetes mellitus, and right-sided stroke were not significantly associated with outcome. Across territories, the proportion of mRS 2 was highest in MCA lesions (60.47%) among clinically meaningful subgroup sizes, although the single frontal case had mRS 2 and should be interpreted cautiously (Table 4, Figure 5).

**Table 4:** Key differences between mRS 2 and mRS 0–1 groups NIHSS: National Institutes of Health Stroke Scale; mRS: modified Rankin Scale; IQR: interquartile range

Variable	mRS 2 (n=45)	mRS 0–1 (n=55)	t-value	p-value
Age, mean ± SD	64.91 ± 7.25	64.76 ± 7.05	0.10	0.919
Lag days, median (IQR)	54 (33–65)	50 (33–64)	-	0.552
NIHSS, mean ± SD	3.80 ± 0.69	3.29 ± 0.76	3.4	0.001
Male sex, n (%)	27 (60.0)	36 (65.5)	-	0.678
Hypertension, n (%)	40 (88.9)	49 (89.1)	-	1.000
Diabetes mellitus, n (%)	26 (57.8)	41 (74.5)	-	0.090
Thalamic stroke, n (%)	7 (15.6)	14 (25.5)	-	0.324

## Discussion

The present study found a CPSP prevalence of 13.9% (100/720) among acute ischemic stroke patients prospectively screened over a two-year period and describes the clinical and radiological profile of CPSP within this affected cohort, highlighting the relationship between lesion distribution, pain laterality, and functional status. Among patients with CPSP, the most frequent lesion pattern involved the MCA territory, followed by thalamic lesions, while pain was contralateral to the stroke lesion in 98% of patients. These findings support the notion that CPSP is a lesion of central somatosensory pathways and not merely a peripheral pain mechanism [20,21]. They are consistent with current evidence that CPSP is associated with disruption of spinothalamic and related thalamocortical networks [22,23].

A review of the literature shows both consensus and important contextual differences. Studies have demonstrated substantial variation in the prevalence of CPSP, with pooled estimates of approximately 11% in the general stroke population [7], but higher rates have been observed in specific lesion subgroups and in studies with more inclusive diagnostic criteria [24,25]. Our prevalence estimate of 13.9% falls within this reported range; however, because our cohort was restricted to patients with mild stroke severity and disability, direct comparison with studies enrolling unselected stroke severity should be made cautiously, and the detailed clinical characterization that follows applies specifically to the symptomatic patients meeting CPSP criteria within this cohort. The frequency of thalamic and extra-thalamic lesions in our cohort is consistent with recent literature describing CPSP as not limited to classical thalamic strokes but also to lesions in cortical, capsular, brainstem, and other somatosensory relay structures [12,13]. The increased prevalence of contralateral pain in the present study is consistent with the classical definition of CPSP as pain in the part of the body corresponding to the damaged central sensory pathway [10].

One of the key findings of this study is that the degree of severity of stroke is associated with functional outcome. Patients with mRS 2 had significantly higher NIHSS scores, and NIHSS was an independent predictor of worse functional status in multivariable analysis. This suggests that the neurological impact of stroke may be more relevant for disability in CPSP than the lesion type itself [26]. This finding aligns with previous reports of post-stroke pain being associated with lower activities of daily living, lower quality of life, lower rehabilitation participation, and higher psychosocial burden [15]. The variation in time lag from stroke onset to symptom evaluation across lesion territories in our cohort corresponds to the finding that CPSP often occurs weeks to months after stroke, resulting in delayed detection during routine follow-up [27].

There are several limitations to the study that should be acknowledged. Although prevalence was calculated from the full screened cohort (100/720, 13.9%), detailed clinical, sensory, and radiological data were retained only for patients meeting CPSP diagnostic criteria. This precludes direct comparison between CPSP-positive and CPSP-negative screened patients, and therefore independent risk factors for the development of CPSP itself - as opposed to predictors of functional outcome within the CPSP-positive group, which the multivariable model does address - cannot be identified from this dataset. In addition, restricting enrollment to patients with mild stroke severity and disability (NIHSS < 5, mRS 0-2) may underestimate the true burden of CPSP, since more severely affected stroke survivors - who may have communication or cognitive impairments limiting self-report of sensory symptoms - were not represented in this cohort. Second, this was a single-center study with a relatively small number of patients, which may limit generalizability and reduce the power for subgroup analyses, particularly for less common lesion sites such as frontal, medullary, and pontine strokes. The diagnosis of CPSP in clinical practice is difficult because of overlap with other post-stroke pain syndromes, and the absence of standardized quantitative sensory testing (e.g., DN4, LANSS) and long-term repeated assessments may have contributed to diagnostic and temporal misclassification [28,29]. Diagnostic classification relied on consensus review among study neurologists rather than a formally quantified interobserver agreement statistic, which limits assessment of diagnostic reproducibility; future studies should incorporate a measure such as Cohen's kappa across independent raters. The follow-up window in this cohort (median 51 days, IQR 33-65) may be insufficient to capture CPSP with delayed onset, which has been reported to emerge months after stroke [27]; therefore, later-onset cases may be underrepresented. Finally, quality of life, mood, sleep disturbance, and treatment response were not assessed in this cohort despite their established relevance to CPSP [15] and should be incorporated into future prospective studies of this condition.

## Conclusions

CPSP affected 13.9% (100/720) of the acute ischemic stroke patients screened in this cohort and is characterized by predominantly contralateral pain, with MCA and thalamic lesions being the most frequent radiological substrates among affected patients. Most patients had mild stroke severity, but a considerable proportion had residual functional impairment at follow-up. Furthermore, a higher NIHSS score was an independent predictor of poor outcome, highlighting the importance of neurological severity as a determinant of post-stroke disability in patients with CPSP.

These results highlight the need for thorough screening of CPSP at routine follow-up after stroke, particularly in patients presenting with sensory symptoms, contralateral pain, and increased initial stroke severity. Timely recognition and successful intervention may optimize rehabilitation strategies and functional recovery. Because this cohort was restricted to patients with mild stroke severity, future large prospective studies enrolling patients with unselected stroke severity are needed to confirm the prevalence estimate, identify independent risk factors for CPSP through comparison with CPSP-negative patients, and define the long-term consequences of CPSP after ischemic stroke.
